# High Intellectual Potential and High Functioning Autism: Clinical and Neurophysiological Features in a Pediatric Sample

**DOI:** 10.3390/brainsci11121607

**Published:** 2021-12-03

**Authors:** Assia Riccioni, Stefano Pro, Lorena Di Criscio, Monica Terribili, Martina Siracusano, Romina Moavero, Massimiliano Valeriani, Luigi Mazzone

**Affiliations:** 1 Child Neurology and Psychiatry Unit, Tor Vergata University Hospital, Fondazione PTV, Oxford Street 81, 00133 Rome, Italy; lorenadiscriscio@live.it (L.D.C.); monica.terribili@gmail.com (M.T.); siracusanomartina@hotmail.it (M.S.); luigi.mazzone@uniroma2.it (L.M.); 2 Systems Medicine Department, University of Rome Tor Vergata, Montpellier Street 1, 00133 Rome, Italy; romina.moavero@opbg.net; 3 Child Neurology Unit, Neuroscience Department, Bambino Gesù Children’s Hospital, IRCCS, Piazza S. Onofrio 4, 00165 Rome, Italy; stefano.pro@opbg.net (S.P.); massimiliano.valeriani@opbg.net (M.V.); 4 Department of Biomedicine and Prevention, University of Rome Tor Vergata, Via Montpellier 1, 00133 Rome, Italy; 5 Center for Sensory Motor Interaction, Aalborg University, 9100 Aalborg, Denmark

**Keywords:** intellectually gifted, autism spectrum disorder, EEG, mismatch negativity, P300, children

## Abstract

High Intellectual Potential (HIP) and High Functioning Autism (HFA) are two different conditions sharing some clinical and neurobiological features. The aim of the present study was to characterize a sample of HIP children (n: 16; M/F: 14/2; median age: 10 years) in comparison to those with HFA (n: 17; M/F: 16/1; median age: 13 years) and to neurotypically developed (NTD) children (n: 10; M/F: 4/6; median age: 11 years) from a clinical and neurophysiological perspective. Specifically, a standardized clinical assessment of cognitive and adaptive skills, autistic symptoms, executive functions and behavioral features was performed. Moreover, event-related potentials (ERPs) were recorded, referring specifically to the mismatch negativity (MMN) and P300 paradigm. Our data highlighted the presence of similarities between the intellectually gifted individuals and the ones with autism (i.e., a nonhomogeneous intellective profile, an adaptive skills impairment, subthreshold autistic symptoms and increased perfectionism). Interestingly, a distinct neurophysiological characterization between groups came out, with evidence of a reduced MMN amplitude only in the HFA group. Furthermore, no differences within groups in the P300 component emerged. Therefore, our results start to provide a more informative characterization of the HIP phenotype in comparison to those of HFA and NTD, highlighting the potential role of the MMN amplitude index in helping clinicians and researchers to distinguish between HIP and HFA. Nevertheless, further research on the topic is strongly needed.

## 1. Introduction

The High Intellectual Potential (HIP) condition refers to individuals who present a Full Scale Intellectual Quotient (IQ) measured by the Wechsler Intelligence Scales for Children [1] above the average (above the 95th percentile) [2,3,4]. It has been described that HIP individuals, commonly named “intellectually gifted” people, generally present a nonhomogeneous cognitive profile, characterized by discrepancies across the factorial indexes of the Wechsler Intelligence Scales. Specifically, HIP individuals mostly demonstrate a reliable accomplishment on the Verbal Comprehension Index (VCI) and a worse performance on the Processing Speed Index (PSI) [5,6,7,8], with a subsequent possible impact on neuropsychological profile [3]. Specifically, even if intellectually gifted individuals generally demonstrate greater attentive and memory abilities in comparison to average cognitive ability peers [9], it has been reported that HIP individuals who present a nonhomogeneous cognitive profile are more likely to demonstrate “fluctuating attentive” skills, meant as the ability of being focused on specific personal interests (e.g., videogames) but not on others (e.g., schoolwork) [4]. From a neurobiological point of view, these findings are supported by the evidence in this population of a more effective inter-hemispheric connectivity with a major involvement of the right cerebral hemisphere [4,10,11,12,13], which is known to be crucial in the control of “selective attention” abilities [14]. Furthermore, a specific pattern of cortical plasticity has been reported, characterized by a progressive reduction of cortical thickness from childhood to adolescence, mainly involving the prefrontal cortex and right superior frontal gyrus [15].

These findings together sustain the hypothesis that intellectually gifted people could present an atypical neurodevelopmental trajectory [3,4]; this in turn could lead, on one hand, to extraordinary skills and, on the other, to difficulties related to a high IQ and the presence of a heterogeneous neurocognitive profile [16]. For example, it is well known that HIP children, paradoxically, can exhibit scholastic difficulties, including school failure, often being mistaken as listless and, at least, not “so smart” [17]. Consequently, HIP individuals could exhibit an increased risk of socio-emotional fragility [3], mostly characterized by difficulties in managing emotions [18,19] and in establishing satisfying social relationships [3,17]. Available data in the field of HIP describe the presence of specific and atypical patterns of interest, as well as a tendency to withdraw, among this population [3,4]. Therefore, it comes out that HIP children could often present clinical and neurobiological features common to other neurodevelopmental disorders—specifically, autism spectrum disorder (ASD) [4,20,21]—making the distinction between them challenging in both, clinical and research activities [22].

As a matter of fact, ASD is a neurodevelopmental condition characterized by an early onset of persistent social and communication difficulties, in addition to a set of restricted and repetitive patterns of interest and behaviors [23], whose clinical expression can vary deeply depending on the autistic symptoms’ severity and adaptive skills impairment [24,25]. Particularly, the term “high-functioning autism” (HFA) refers to autistic people who present an IQ value equal or above 70 with no severe impairment of adaptive and language abilities [26]. Just like HIP individuals, HFA subjects often present a heterogeneous cognitive profile on the Wechsler Intelligence Scales [27], including executive function deficits [28], in addition to difficulties with emotional regulation and social skills [24,29]. Moreover, an inter-hemispheric hyper-connectivity with an unbalanced neurological lateralization, as well as an atypical cortical thickness, has been described in autistic individuals [24,30,31].

Despite evidence of cognitive and developmental similarities between HIP and HFA [4,20,21,22,32] and the growing interest in evaluating such aspects [26], to the best of our knowledge, only few data are available from empirical studies [4,33]. Accordingly, Boschi et al. [4], in a systematic review on the topic, highlighted the urgent need for further investigations aimed at better describing similarities and differences between HIP and HFA, not only on the basis of neurocognitive profiles and intellective performances, but also on a more comprehensive psychophysiological assessment. Findings emerging from these studies might lead to a better understanding of both HIP and HFA, with subsequent possible important implications in terms of differential diagnosis, clinical prognosis and therapeutic strategies.

In such context, neurophysiological techniques such as event-related potentials (ERPs), could play a role. ERPs represent a non-invasive method broadly investigated in neuropsychiatric research activities [34] to evaluate neurocognitive and attentive processes. Among ERPs, the mismatch negativity (MMN) and the P300 component have been widely explored in neurodevelopmental disorders [35,36,37,38,39] including autism [40,41,42,43].

Specifically, the mismatch negativity (MMN) index is a negative wave localized in the fronto-central brain regions [44] arising from the auditory and frontal cortex ~100–200 ms after the onset of infrequent stimuli (“deviants”) intermingled in a series of repetitive stimuli (“standards”) [35]. MMN generally reflects an automatic passive cerebral discrimination process, without attentive control, thus involving cognitive processing [45,46] and executive function abilities (i.e., “set-shifting” ability and working memory) [47,48,49]. P300 is a positive wave that is automatically raised after the MMN waveform ~300 ms following a stimulus. P300 generally reflects attentional and executive function abilities. It is closely linked to cognitive functions and broadly recognized as a sensitive marker of intellectual impairment [50,51,52]. Available data reported ERP abnormalities in ASD [41,42,53] as well as HIP individuals [2,54], mainly characterized by an increased amplitude and reduced latency on MMN indexes and altered P300 amplitude. Nevertheless, to the present date, no previous studies have investigated differences in ERP indexes between HIP and HFA and the correlation to clinical features.

Thus, given the lack of empirical studies and the growing interest in the topic, the aim of the present study was to characterize a pediatric sample of HIP individuals in comparison to a group of HFA individuals and a neurotypical developmental (NTD) control group —not only from a clinical perspective, but also from a neurophysiological point of view, in order to better describe the HIP phenotype in comparison to HFA and NTD children.

## 2. Materials and Methods

The present study was approved by the Ethical Committee of our university hospital, the Fondazione Policlinico Tor Vergata (register number 126/18, June 2018), and informed consent was obtained from all legal holders of custody of all included individuals.

In particular, the Child Psychiatry Unit of the University of Rome Tor Vergata Hospital was responsible for the sample’s recruitment and the clinical assessment, whereas the Child Neurology Unit of the Bambino Gesù Children’s Hospital of Rome was the representative for the neurophysiological recordings and the data and statistical analysis procedure.

### 2.1. Participants

Our sample constituted children (age range 6–16 years) recruited from the Child Psychiatry Unit of the University of Rome Tor Vergata Hospital between January 2019 and January 2020. Specifically, the participants included in the present study were assessed for their eligibility by a multidisciplinary team (child psychiatrists and psychologists).

In order to be eligible, participants were required to have: (1) a condition of High Intellectual Potential (HIP), defined as the presence of an Intelligence Quotient (IQ) assessed by the Wechsler scale on the average; (2) a diagnosis of autism spectrum disorder without language and/or cognitive impairment (IQ above 70) on the basis of the Diagnostic and Statistical Manual of Mental Disorders–Fifth Edition (DSM–5) criteria [23], supported by the application of the Autism Diagnostic Observation Schedule–Second Edition (ADOS–2) [55,56]. By contrast, individuals with other neurological or psychiatric associated conditions (i.e., epilepsy, attention deficit and hyperactivity disorder or auditory deficit) were excluded.

Moreover, a control group of neurotypical developmental (NTD) individuals (age range 6–16 years) was included, voluntarily recruited from a sport club.

A final sample of 43 individuals was involved, divided into three groups: HIP (n: 16; M/F: 14/2; age M +/− SD: 10.12 (2.28)); HFA (n: 17; M/F: 16/1; age M +/− SD: 13.17 (2.35)); and NTD (n: 10; M/F: 4/6; age M +/− SD: 10.8 (3.93)).

### 2.2. Procedure

The HIP and HFA groups underwent a comprehensive standardized clinical assessment of cognitive abilities, adaptive skills, autistic symptoms, executive functions and behavioral aspects, as described below.

The NTD group performed a clinical screening evaluation of: IQ (based on age: Raven’s Colored Matrices for age < 11 years, or Raven’s Progressive Matrices for age > 11 years); behavioral problems (Conners’ Parent Rating Scale-Revised, CPRS-R [57]); autistic symptoms (Autism Diagnostic Observation Schedule- Second Edition, ADOS-2 [55]; Social Responsiveness Scale, SRS [58]). All NTD individuals were negative for the presence of cognitive impairment (IQ assessed with Raven’s Matrices above the 25th–50th centile for the colored form and above 85 for the progressive form), for any behavioral problems and for the presence of autistic symptoms.

Finally, the whole sample (HIP, HFA and NTD) underwent an electroencephalogram, specifically aimed to evaluate the MMN and the P300 indexes.

### 2.3. Clinical Assessment

#### 2.3.1. Cognitive Abilities

The Wechsler Intelligence Scale for Children–Fourth Edition (WISC–IV) [1] was applied to both HIP and HFA groups. The WISC-IV is an intelligence test for children aged from 6 to 16 years. It provides five main cognitive ability scores (verbal comprehension index, VCI; perceptual reasoning index, PRI; working memory index WMI; processing speed index, PSI) and a Full Scale Intelligence Quotient (IQ). Each of these indexes is set to have a mean of 100 and a standard deviation of 15.

#### 2.3.2. Adaptive Skills

The Adaptive Behavior Assessment System–Second Edition (ABAS–II) [59], was applied to all HIP and HFA parents. The ABAS–II is a parent-report questionnaire that provides a measurement of children’s skills relating to their development, behavior and cognitive abilities. In particular, the “5–21 years” ABAS–II form was used. Parents were asked to rate their child’s skills at completing an activity (from 0 = “not able to” to 3 = “able to do it and always performs it when needed”) in regards to 10 functioning areas (i.e., communication, use of environment, preschool competences, domestic behavior, health and safety, play, self-care, self-control, social abilities and motility). The questionnaire provides three main adaptive domains: conceptual (CAD), practical (PAD), social (SAD) and a comprehensive score (General Adaptive Composite, GAC). Each of these indexes is set to have a mean of 100 and a standard deviation of 15.

#### 2.3.3. Autistic Symptoms Assessment

All participants underwent the ADOS–2 test (Autism Diagnostic Observation Schedule–Second Edition, ADOS–2) [55], performed by a licensed clinician. The ADOS–2 is a semi-structured observational evaluation of autistic symptoms, including five modules based on the subject’s expressive language level and age. The ADOS–2 algorithm is organized by social affect (SA), restricted and repetitive behaviors (RRB) and total score (TOT). Modules 1, 2 and 3 provide the calibrated severity score (CSS), ranging from 1 to 10, indicating a measure of the subject’s autism severity level. In the present study based on age and language skills, Module 3 was applied to all participants.

Moreover, the social responsiveness scale (SRS) [58] was performed. The SRS is a 65-item questionnaire applied to parents of children aged between 4 and 18 years. The SRS consists of five subscales based on diagnostic criteria for ASD: social motivation, social awareness, social cognition; social communication; and restricted interests and repetitive behavior. Total scores can be converted into T-scores in order to give an indication of severity for an individual’s symptoms. T-scores falling within the mild, moderate or severe range suggest clinically significant symptoms with varying degrees of impact on everyday social interactions.

#### 2.3.4. Neuropsychological Assessment

The NEPSY–Second Edition (NEPSY–II) [60] is a comprehensive battery of tests widely utilized to assess the neuropsychological development of children aged between 3 and 16 years old. The NEPSY–II consists of different subtests that can be used in various combinations. In the present study, the “auditory attention”,”visual attention”, “response set”, “design fluency” and “inhibition” items were applied in order to evaluate the attention and executive functioning domains. Specifically, the subcomponents of attention and executive functioning that were assessed include the inhibition of learned and automatic responses, vigilance and self-regulation, selective and sustained attention, as well as set shifting abilities.

#### 2.3.5. Behavioral Problems Evaluation

The Conners’ Parent Rating Scale–Revised (CPRS–R) [57] is a parent-report questionnaire aimed at evaluating behavioral difficulties during childhood and adolescence, referring specifically to symptoms of Attention and Hyperactivity Deficit Disorder (ADHD) such as hyperactivity and inattention. Specifically, parents are asked to rate their child’s behavior on a four-point Likert Scale (0 = not true at all; 1 = just a little true; 2 = pretty much true; 3 = very much true). The “long form” consists of 80 items grouped into 8 subscales (cognitive problems, oppositional, hyperactivity/impulsivity, anxious/shy, perfectionism, social problems and psychosomatic). Furthermore, the scale provides an ADHD Index score, which enables the detection of children at risk of Attention Deficit and Hyperactivity Disorder. According to the T-scores, the behavior is considered as typical (T < 60), borderline (T = 61–69), or clinically significant (T ≥ 70). Particularly, the psychometric properties demonstrated good reliability coefficients and a high test–retest reliability, as well as a good discriminatory power [57].

### 2.4. Neurophysiological Recording

For the ERP recording, the Micromed Brain Quick System Plus (Micromed, Mogliano Veneto, Italy) was used. Subjects were comfortably seated in a quiet room. Mismatch negativity (MMN) recording preceded the P300 recording of all our children and adolescents. Auditory stimuli were sinusoidal tones (10 ms duration, 2 ms rise time, 2 ms fall time and 85 dB SPL of intensity) presented binaurally via headphones. Frequent 750 Hz tones and deviant 500 Hz tones were delivered with a probability of 85% and 15%, respectively. A fixed interstimulus interval (ISI) of 1 s and an ISI variable between 0.8 and 1.2 s were used, respectively, for MMN and P300 recording. Event-related potentials were recorded from three scalp electrodes, located at the Fz, Cz and Pz positions of the 10–20 International System. A further electrode placed in the outer cantus of the right eye recorded the electro-oculogram (EOG). The reference was at the nose. The electroencephalogram (EEG) sampling rate was 1024 Hz, and the analysis time was 1000 ms, including 100 ms of prestimulus delay. The amplifier bandpass was 0.1 to 30 Hz (24 dB roll-off). An automatic artifact rejection system excluded from the average all runs containing transients exceeding ±150 µV at any recording channel, including the EOG. Averages of 15 trials (deviant stimuli) were used for ERP measurements.

#### 2.4.1. MMN Recording

Mismatch negativity was recorded after 100 acoustic stimuli. Children were instructed to read a novel; thus, they did not pay attention to the acoustic stimulation. They were required to summarize the novel in a short briefing following the stimulation.

#### 2.4.2. P300 Recording

Children underwent a block of ∼100 acoustic stimuli. They were instructed to count the number of infrequent tones mentally. No motor response was required. Averages in which counting mistakes had exceeded 10% would not have been considered in the data analysis.

#### 2.4.3. ERPs Analysis

The N1 and P2 latencies and the peak-to-peak N2/P2 amplitude were measured in the Cz traces recorded to deviant stimuli. For MMN labeling, difference traces were calculated by subtracting the frequent stimuli from the deviant stimuli traces. In the Fz difference trace, the MMN latency and amplitude were measured at the peak and from the baseline, respectively. The P300 latency and amplitude were measured in the Pz trace to deviant stimuli, respectively at the peak and from the baseline.

## 3. Statistical Analysis

Clinical and socio-demographic data were presented as means, SDs and frequencies (percentages).

Differences between groups (HFA vs. HIP vs. NTD) were investigated using one-way analysis of variance (ANOVA), Student’s *t*-test and Pearson’s chi-square test (χ^2^). Finally, an explorative correlation analysis was performed in order to investigate the relation between MMN and clinical indexes. Statistical significance was set at *p-*values < 0.05.

## 4. Results

### 4.1. Demographic and Clinical Data

#### 4.1.1. Cognitive and Adaptive Functioning Profiles

Different cognitive profiles were found in the HIP and HFA groups. Specifically, the HIP group exhibited higher scores in all WISC–IV cognitive indexes (HIP vs. HFA: *VCI p* = 0.002; PRI *p* < 0.001; WMI *p* < 0.001; PSI *p* = 0.023), and greater full IQ scores (IQ *p* < 0.001), in comparison to the HFA group. However, particularly with respect to each WISC–IV factorial index (VCI, PRI, WMI, PSI), both groups (HIP and HFA) showed lower than average scores on the WMI and PSI index values (M ± SD WMI, HIP: 111.9 ± 15.9, HFA: 91.1 ± 11.8; PSI, HIP: 106.4 ± 15.7, HFA: 93.6 ± 15.1) in comparison to those obtained on the VCI and PRI indexes (Table 1).

Moreover, when analyzing the adaptive functioning profile, the HIP group exhibited greater abilities in all ABAS–II domains in comparison to the HFA group (GAC *p* < 0.001; CAD *p* < 0.001; SAD *p* = 0.014; PAD *p* = 0.008). However, when comparing the HIP group to the NTD ones, a statistically significant difference came out in the GAC, SAD and PAD domains (GAC *p* = 0.010; SAD *p* = 0.020; PAD *p* = 0.004) although not in the CAD domain (*p* = 0.205), meaning that the HIP individuals presented conceptual adaptive domain values that were better than the HFA patients and similar to the NTD subjects (Table 1).

#### 4.1.2. Autistic Symptoms

Different ADOS–2 CSS and SRS scores were found in different groups. Specifically, when comparing the HIP individuals to the HFA group, statistically significant differences in terms of ADOS–2 CSS and SRS scores (ADOS–2 CSS HIP vs. HFA *p* < 0.001; SRS total score HIP vs. HFA *p* = 0.007) were observed, with the HIP group showing a lower level of autistic symptoms in comparison to the HFA one. However, when comparing the HIP group to the NTD, a significant difference in terms of ADOS–2 CSS scores emerged (ADOS–2 CSS HIP vs. NTD *p* = 0.048), with a trend of higher level of autistic symptoms in the HIP group. Interestingly, no significant differences were found within the SRS scores (SRS total score HIP vs. NTD *p* = 0.244) (Table 1).

#### 4.1.3. Executive Functions and Behavioral Profile

Compared to HFA individuals, the HIP group showed a different performance in the NEPSY–II (Table 2). Particularly, the HIP individuals presented higher NEPSY–II scores on the inhibition and response set (HIP vs. HFA: Inhib *p* < 0.001; RS *p* < 0.001), as well as on the visual attention items (HIP vs. HFA *p* = 0.006). However, no significant differences were found for the design fluency (*p* = 0.307) and auditory attention (*p* = 0.099) items. In regards to the CPRS scores, the HFA group showed higher values for the anxiety/shyness (A/S) and social problems (SP) items when compared to the HIP group (HIP vs. HFA: A/S *p* = 0.020; SP *p* = 0.047) and to the NTD group (HFA vs. NTD: A/S *p* < 0.001; SP *p* = 0.031). Both HIP and HFA groups had greater scores for the perfectionism item than NTD individuals (HIP vs. NTD *p* < 0.001; HFA vs. NTD *p* = 0.001) (Table 2).

### 4.2. MMN Parameters

**Figure 1:**
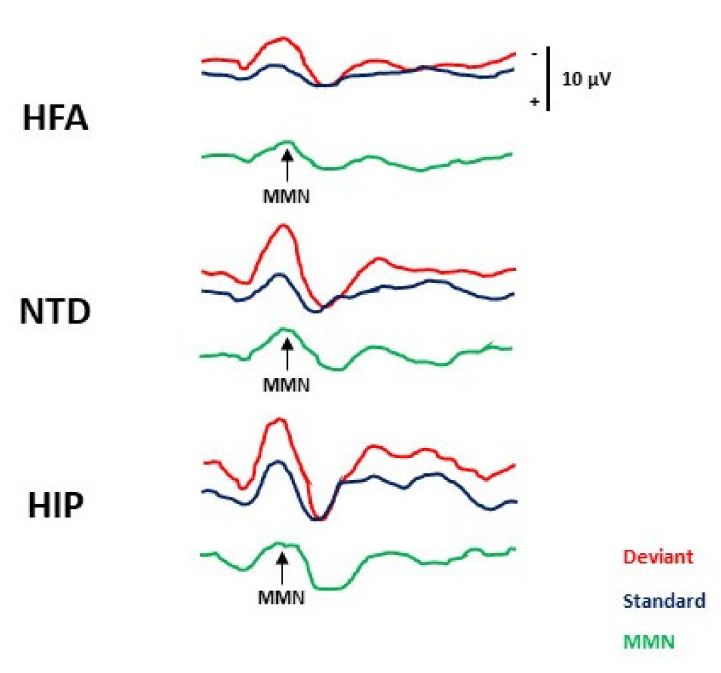
MMN waveforms in HFA, NTD and HIP individuals. The figure shows ERP traces (Fz) recorded in representative HFA (upper), NTD (middle) and HIP (lower) subjects. ERPs to standard and deviant stimuli are shown in blue and red, respectively. Green traces are calculated off-line by subtracting the traces to standard stimuli from those to deviant stimuli. The analysis time was 500 ms. A clear MMN (arrow) component is identifiable in the subtraction traces.

ANOVA did not provide significant differences in the MMN latency within the three groups (HIP M ± SD 94.6 (28.6) vs. HFA 93.96 (20.08) vs. NTD 83.61 (25.5); HIP vs. HFA *p* = 0.940; HFA vs. NTD *p* = 0.245; HIP vs. NTD *p* = 0.200) (Table 3, Figure 1).

By contrast, the MMN amplitude varied between the groups. Specifically, HFA patients showed a reduced MMN amplitude compared to HIP patients (HFA vs. HIP: *M ± SD* 4.45 (1.13) vs. *M ± SD* 6.39 (2.66); *p = 0.001*) and to NTD (*M ± SD* 6.37 (1.84); *p < 0.001*) subjects. No significant difference was found between the HIP and the NTD group (*p* = 0.99) (Table 3, Figure 1).

### 4.3. P300 Parameters

No significant differences came out within groups in terms of P300 values (HIP vs. HFA *p* = 0.347; HIP vs. NTD *p* = 0.420; HFA vs. NTD *p* = 0.616) (Table 3).

### 4.4. Correlation between MMN-Component Characteristics and Clinical Data

Correlation coefficients, with Bonferroni adjusted *p*-values were computed between the MMN amplitude and latency and all clinical variables.

No significant correlations came out between the MMN parameters and cognitive indexes as well as the adaptive functioning parameters. Moreover, no significant results emerged when comparing the MMN indexes to ADOS–2 and CBCL scores.

However, our data demonstrated a positive correlation between MMN latency values and the SRS social cognition scores (*r =* 0.53; *p* = 0.035; *R*^2^ = 0.280) only among the HIP population, meaning that a worse social cognition performance (defined as a greater SRS_SC score) is related to an increased MMN latency in this group. No significant correlation came out in the HFA (*r* = 0.038; *p =* 0.88; *R*^2^ *=* 0.002) (see Supplementary Material, Table S1).

Finally, in the HFA group we found a positive correlation between the perfectionism index assessed by the CPRS parent’s questionnaire and the MMN latency values (*r =* 0.54; *p =* 0.023; *R*^2^ *=* 0.297) and a negative correlation between NEPSY–II visual attention scores and MMN latencies indexes (*r* = −0.62; *p =* 0.007; *R*^2^ *=* 0.386) (see Supplementary Materials, Table S1).

## 5. Discussion

To the best of our knowledge, the present study is the first aimed at characterizing, from a clinical and neurophysiological perspective, a pediatric sample of HIP individuals compared to those with HFA and NTD subjects.

In line with available data [33], our results demonstrated that intellectually gifted individuals show better cognitive performances, greater IQ values and superior adaptive skills in comparison to those with HFA. In such a context, Doobay et al. [33], in a work on a sample of 40 HFA and 41 HIP subjects (aged 5–17 years), described better social abilities as well as cognitive and adaptive skills in the HIP group, stating that evidence of adaptive functioning impairment in the intellectually gifted population is not available. However, in this study the lack of NTD individuals prevented us from reaching definitive conclusions. Instead, in our study a worse adaptive functioning in the intellectually gifted population (especially regarding the practical and social domains) was found in the comparison to HIP and HFA.

With a particular focus on social skills, it is well known that HIP children may often show difficulties in the social functioning profile [17,18,19]. However, to our knowledge, this aspect was not previously investigated within this population by standardized gold-standard measures, such as the ADOS–2 and the SRS questionnaire, in comparison to both autistic and neurotypical children. In such a context, our data pointed out the presence of subthreshold autistic symptoms assessed by the ADOS–2 in the intellectually gifted subjects when compared to NTD ones. Moreover, even if HIP children generally demonstrated greater scores on the NEPSY–II battery items in comparison to those with HFA, no differences within groups were found on the *set-shifting* abilities, particularly referred to the attention auditory and design fluency items. Finally, as a behavioral characteristic, our results highlighted the presence of increased perfectionism in the HFA and the HIP children. Based on the knowledge that *set-shifting* abilities are a general marker of cognitive flexibility [61], which in turn is correlated with perfectionism [62], we may speculate that our results could reflect the presence of intellectual rigidity not only in the autistic but also in the intellectually gifted children, with consequent possible influence on neuro-cognitive performances.

As the mismatch negativity (MMN) and P300 component are reliable markers of neuro-cognitive mechanisms [35,36,44,63], we evaluated whether a specific neurophysiological pattern could be found within groups.

Interestingly, no differences on P300 indexes came out. Our data stand in contrast with previous available studies, showing an increased amplitude and reduced latency of P300 in HIP individuals when compared to intellectually average peers [2,54]. However, it is important to take into account that only few studies are available on the topic, mainly concerning small samples. As the P300 latency and amplitude values are closely related to IQ [50,51,52,64], we may hypothesize that our results could be influenced by the absence of cognitive impairment in all our included subjects. Nevertheless, further investigations on the topic are needed.

By contrast, in line with previous studies [41,42,43] we found a reduced MMN amplitude in the autistic population. However, no significant abnormalities emerged in the HIP group, which showed a mean MMN amplitude similar to that of NTD subjects. Our results stand in contrast with available data [2,54], reporting differences in the MMN between HIP and cognitive average individuals. Particularly, Liu et al. [2], investigating the relationship between MMN and intelligence in a sample of 18 intellectually gifted children compared to 18 NTD (mean age 11.8), found an increased amplitude and a decreased latency in the MMN in the HIP children. Nonetheless, the authors did not exclude the presence of possible concomitant conditions frequently described in HIP children, such as Attention Deficit and Hyperactivity Disorders (ADHD) and autism spectrum disorder (ASD) [3], with a subsequent possible impact on the reported results. In the present study, a concomitant diagnosis of autism and/or ADHD in all HIP subjects was excluded by the use of standardized measures (ADOS–2, SRS and CPRS). Therefore, our data could preliminarily start to support the potential role of the MMN amplitude recording in helping clinicians and researchers to differentiate between high intelligence and autistic subjects from a neurophysiological perspective.

Within this framework, to better investigate differences between the three groups (HIP, HFA and NTD) we analyzed the correlations between MMN indexes and selected clinical features, with a particular focus on the social and behavioral profiles. Interestingly, in the HIP group we found a positive correlation between MMN latency indexes and the social cognition ability reported by parents in the SRS questionnaire, meaning that a worse social cognition (defined as a greater score on the SRS) is related to an increased MMN latency in such individuals. Even if in the HIP population the MMN latency indexes as well as the SRS scores do not reach a significant level, we may speculate that an increased level of anxiety, related to a greater impairment in social cognition, may have had an impact on the MMN latency parameters. Accordingly, in the HFA group we found a positive correlation between the presence of perfectionism and the MMN latency values. Even if our study presents some strengths, such as the well clinically described sample, the presence of a replicable methodological procedure, as well as the inclusion of both HFA and NTD in comparison to HIP individuals, it also exhibits several limitations that should be taken into account. Specifically, the small sample size and the relatively large age range (6–16 years), as well as the differences within groups in terms of mean age and male/female ratio, could represent a bias when interpreting our data.

In conclusion, even if from a clinical point of view our preliminary results confirmed the presence of similarities within the intellectually gifted individuals and the ones with autism (a nonhomogeneous intellective profile, subthreshold autistic symptoms and increased perfectionism), from a neurophysiological perspective a distinct characterization within groups came out. As a result, neurophysiological techniques, specifically referring to the MMN component, could further help clinicians and researchers in distinguishing between HIP and HFA. Nevertheless, the lack of studies on the topic make it difficult to compare our data to the available literature, strongly highlighting the need for empirical investigations on the topic.

## Figures and Tables

**Table 1:** Cognitive performances, adaptive skills and autistic symptoms measures in HIP, HFA and NTD.

	HIP (n: 16)		HFA (n: 17)		NTD (n: 10)		HIP vs. HFA	HFA vs. NTD	HIP vs. NTD
	MEAN	SD	MEAN	SD	MEAN	SD	*p* Value Cohen’s d	*p* Value Cohen’s d	*p* Value Cohen’s d
WISC–IV									
IQ	131.1	8.5	107.1	15.1	-	-	<0.001 1.96	*-*	*-*
VCI	133.2	13.2	114.9	17.8	-	-	0.002 1.17	*-*	*-*
PRI	131.5	10.6	110.1	19.9	-	-	<0.001 1.34	*-*	*-*
WMI	111.9	15.9	91.1	11.8	-	-	<0.001 1.48	*-*	*-*
PSI	106.4	15.7	93.6	15.1	-	-	0.023 0.83	*-*	*-*
ABAS–II									
ABAS_GAC	96.06	14.48	77.47	18.87	110.7	8.47	<0.001 1.10	<0.001 2.27	0.010 1.23
ABAS_CAD	100.1	11.44	83.82	13.66	108.2	12.53	<0.001 1.29	0.003 1.86	0.205 0.67
ABAS_SAD	96.63	17.47	81.06	16.69	111.3	9.28	0.014 0.91	<0.001 2.24	0.020 1.05
ABAS_PAD	92.81	12.67	76.24	20.4	113.3	11.2	0.008 0.97	<0.001 2.25	0.004 1.71
ADOS–2									
ADOS_SA	2.62	2.27	8.29	2.69	0.8	1.30	<0.001 2.28	<0.001 3.54	0.044 0.98
ADOS_RRB	0.75	1.06	1.94	1.29	0	0	0.007 −1.01	<0.001 2.13	0.013 1
ADOS_CSS	1.94	1.44	6.17	1.70	0.8	0.83	<0.001 2.69	<0.001 4.01	0.048 0.97
SRS									
SRS_T	58.38	18.34	75.82	16.65	49	14.91	0.007 0.99	0.004 1.69	0.244 0.56
SRS_SA	50.56	20.72	63.88	11.94	51.33	19.47	0.034 −0.78	0.185 0.77	0.937 −0.04
SRS_SC	48.25	19.01	69.65	15.54	50	13.77	<0.001 1.23	0.016 1.34	0.816 0.10
SRS_SCo	54.13	19.04	74.35	14.08	53	14.6	0.002 1.20	0.013 1.49	0.885 0.07
SRS_SM	50.19	20.61	69.06	14.08	48.67	12.01	0.005 1.07	0.006 1.55	0.833 0.09
SRS_AM	52.25	20.68	74.12	13.2	46.67	11.43	<0.001 −1.26	<0.001 2.22	0.434 0.33

**Legend:** WISC–IV: Wechsler Intelligence Scale for Children–Fourth Edition. IQ: Intelligent Quotient. VCI: Verbal Comprehension Index. PRI: Perceptual Reasoning Index. WMI: Working Memory Index. PSI: Processing Speed Index. ABAS–II: Adaptive Behavior Assessment System–Second Edition. ABAS_GAC: ABAS General Adaptive Domain. ABAS_CAD: ABAS Conceptual Adaptive Domain. ABAS_SAD: ABAS Social Adaptive Domain. ABAS_PAD: ABAS Practical Adaptive domain. ADOS–2: Autism Diagnostic Observation Schedule–Second Edition. ADOS_SA: Social Affect. ADOS_RRB: Restricted and Repetitive Behaviors. ADOS_CSS: Calibrated Severity Score. SRS: Social Responsiveness Scale. SRS_T: SRS Total Score. SRS_SA: SRS social awareness. SRS_SC: SRS social cognition. SRS_SCo: SRS social communication. SRS_SM: SRS social motivation. SRS_AM: SRS autistic mannerism.

**Table 2:** Neuropsychological and behavioral assessment in HIP, HFA and NTD.

	HIP (n: 16)		HFA (n: 17)		NTD (n: 10)		HIP vs. HFA	HFA vs. NTD	HIP vs. NTD
	MEAN	SD	MEAN	SD	MEAN	SD	*p* Value Cohen’s d	*p* Value Cohen’s d	*p* Value Cohen’s d
CPRS–R									
Oppositional	63.19	14.69	64.19	13.76	63.5	14.63	0.843 −0.07	0.923 0	0.965 −0.05
Cognitive pr	56.25	11.25	61.25	13.11	59.33	10.91	0.256 −0.04	0.735 0.16	0.572 −0.27
Hyper/imp	62.06	10.85	61.5	12.47	60.67	11.99	0.892 0.05	0.888 0.07	0.809 0.12
Anx/shy	51.94	11.91	63.63	14.89	47.17	2.99	0.020 −0.87	<0.001 1.53	0.154 0.55
Perfectionism	59	13.42	60.13	16.93	42.83	5.60	0.836 −0.07	0.001 1.37	<0.001 1.57
Social probl	59.31	15.38	72.31	19.82	55.83	11.94	0.047 −0.73	0.031 0	0.585 1.00
Psychosomatic	54.31	8.72	52.63	11.48	51	14.86	0.643 0.16	0.815 0.12	0.624 0.27
ADHD Index	59	11.81	63.50	13.46	60.33	12.11	0.322 −0.35	0.608 0.25	0.822 −0.11
DSM IV_Tot	61.38	11.43	61.94	12.41	62.50	12.74	0.894 −0.05	0.928 0.04	0.854 0.09
NEPSY–II									
Design fluency	9.12	2.33	5.64	3.14		-	0.307 1.26	-	-
Visual attention	11.81	3.56	8.06	3.78			0.006 1.02		
Inhibition	9.87	2.57	6.30	3.12		-	<0.001 1.25	-	-
Auditory attention	4.62	1.40	3.82	1.29		-	0.099 0.60	-	-
Response set	5.25	1.23	3.12	1.70		-	<0.001 1.43	-	-

**Legend:** CPRS-R: Conners’ Parent Rating Scale–Revised. Cognitive pr: cognitive problems. Hyper/imp: hyperactivity-impulsivity. Anx/shy: anxiousness/shyness. Social probl: social problems. DSM IV_Tot: DSM–IV Total score. NEPSY–II: NEPSY–Second Edition.

**Table 3:** Mismatch Negativity (MMN) and P300 component characteristics in HIP, HFA and NTD.

	HIP	HFA	NTD	HIP vs. HFA	HFA vs. NTD	HIP vs. NTD
				*p* Value Cohen’s d	*p* Value Cohen’s d	*p* Value Cohen’s d
MMN amplitude Means (and SDs)	6.39 (2.65)	4.45 (1.13)	6.37 (1.84)	0.001 0.95	<0.001 −1.26	0.99 0.01
MMN latency Means (and SDs)	94.61(28.6)	93.96(20.08)	83.61(25.5)	0.940 0.02	0.245 0.45	0.200 0.40
P300 (MS)	301.88	311.33	306.53	0.347	0.616	0.420

**Legend:** SDs: standard deviations. MS: milliseconds.

## Data Availability

The data presented in this study are contained within the article.

## Supplementary Materials

The following are available online at https://www.mdpi.com/article/10.3390/brainsci11121607/s1, Figure S1: **a**. Electrodes position, **b**. Event Related Potentials (ERPs) protocol. Table S1: Results of correlation analysis between MMN latency indices and SRS, CPRS and NEPSY-II scores within HIP and HFA groups
