# Supplementary material for: High Intellectual Potential and High Functioning Autism: Clinical and Neurophysiological Features in a Pediatric Sample
Source: Brain Sci. 2021 Dec 3;11(12):1607. doi: 10.3390/brainsci11121607 (PMC8699491; doi:10.3390/brainsci11121607)
Supplement: Supplementary file 1 [file brainsci-11-01607-s001.zip › Supplementary materials/Table S1_Rev.pdf]

Table S1: Results of correlation analysis between MMN latency indices and SRS, CPRS and NEPSY-II scores within HIP and HFA groups.

	MMN latency	
	**HIP**	**HFA**
**SRS**		
SRS_T	r= -0.265 p=.320	r= 0.039 p=.881
SRS_SA	r= 0.453 p=.077	r= -0.071 p=.787
SRS_SC	***r= 0.530 p=.035***	r= -0.038 p=.886
SRS_SCo	r= -0.435 p=.091	r= -0.019 p=.942
SRS_SM	r= -0.432 p=.093	r= -0.072 p=.782
SRS_AM	r= -0.326 p=.217	r= -0.236 p=.509
**CPRS-R**		
Oppositional	r= -0.452 p=.078	r= -0.313 p=.221
Cognitive Pr	r= -0.013 p=.961	r= -0.040 p=.876
Hyper/Imp	r= 0.118 p=.663	r= 0.250 p=.333
Anx/Shy	r= -0.378 p=.149	r= -0.284 p=.269
Perfectionism	r= -0.256 p=.337	***r= 0.545 p=.023***
Social Probl	r= -0.218 p=.416	r= -0.217 p=.401
Psychosomatic	r= -0.476 p=.062	r= 0.480 p=.051
ADHD Index	r= 0.235 p=.380	r= -0.030 p=.911
DSM IV_Tot	r= 0.144 p=.530	r= 0.050 p= .847
NEPSY-II		
Design Fluency	r=0.188	r=0.008

	p=.483	p=.974
Inhibition	r=0.166<br>p=.537	r=-0.455<br>p=.053
Visual Attention	r= 0.251<br>p=.0.346	**r=-0.621**<br>**p=.007**

Legend:

SRS: Social Responsiveness Scale
SRS_T: SRS Total Score
SRS_SA: SRS social awareness
SRS_SC: SRS social cognition
SRS_SCo: SRS social communication
SRS_SM: SRS social motivation
SRS_AM: SRS autistic mannerism
CPRS-R: Conners' Parent Rating Scale – Revised
Cognitive Pr: Cognitove Problems
Hyper/Imp: Hyperactivity-Impulsivity
Anx/Shy: Anxious-Shyness
Social Probl: Social Problems
DSM IV_Tot: DSM-IV Total score
