# Supplementary figures and images for: High Intellectual Potential and High Functioning Autism: Clinical and Neurophysiological Features in a Pediatric Sample
Source: Brain Sci. 2021 Dec 3;11(12):1607. doi: 10.3390/brainsci11121607 (PMC8699491; doi:10.3390/brainsci11121607)

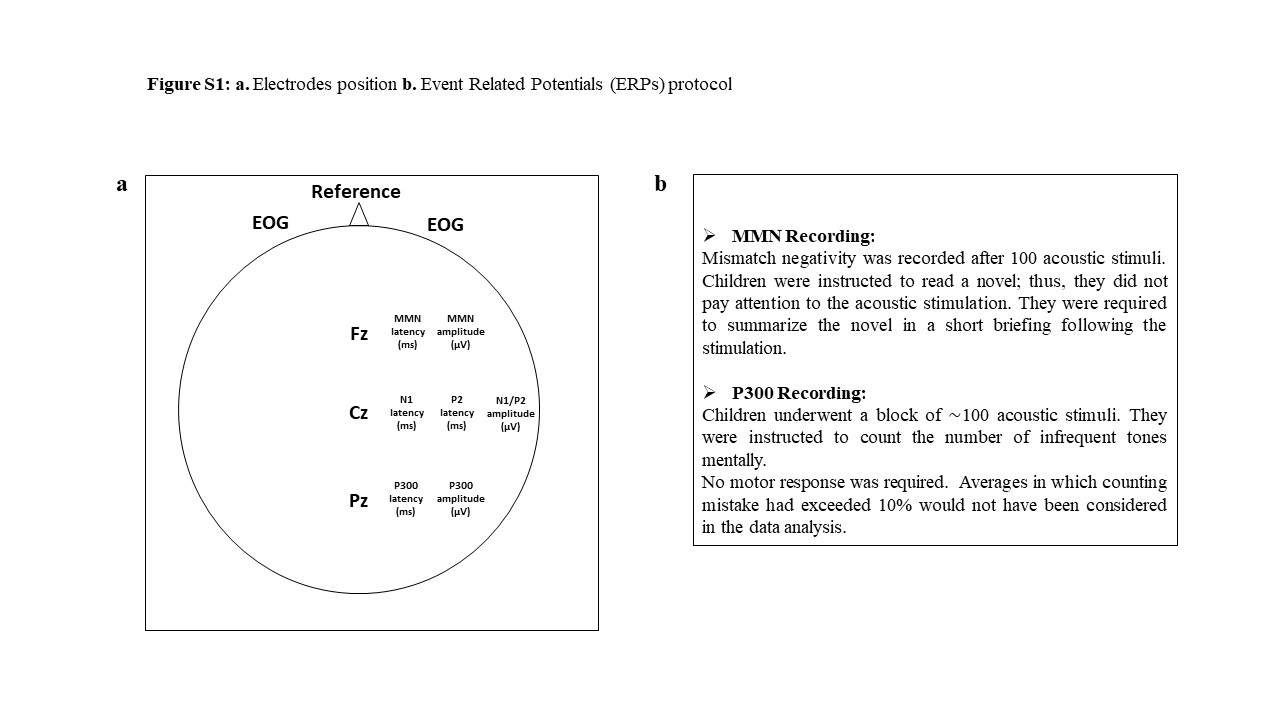

Supplement: Supplementary file 1 [file brainsci-11-01607-s001.zip › Supplementary materials/Figure S1_ ERPs protocol.jpg]
